# The swine market movement and structure in Mexico: Implications for animal welfare and environmental impact

**DOI:** 10.1371/journal.pone.0327469

**Published:** 2025-07-09

**Authors:** Nicolás Callejas-Juárez, Francisco Ernesto Martínez-Castañeda, Nathaniel Alec Rogers-Montoya, Elein Hernandez

**Affiliations:** 1 Universidad Autónoma del Estado de Chihuahua. Facultad de Zootecnia y Ecología, Chihuahua, México; 2 Universidad Autónoma del Estado de México. Instituto de Ciencias Agropecuarias y Rurales, Toluca, México; 3 Colegio de Postgraduados. Programa de Ganadería. Campus Montecillo, Texcoco, México; 4 Universidad Nacional Autónoma de México. Department of Clinical Studies and Surgery, Facultad de Estudios Superiores Cuautitlán; Nanyang Technological University, SINGAPORE

## Abstract

The aim of this study was to analyze the evolution of Mexico’s swine movement network over a five-year period (2017–2021), using a network approach to examine the significant implications of pig movement on animal welfare and environmental impact. Swine-movement records used were obtained from the Servicio Nacional de Sanidad, Inocuidad y Calidad Agroalimentaria (SENASICA). In the period 2017–2021, 33.3 million pigs were transported each year, 87.29% of them for slaughter, 12.20% for fattening, 0.50% for breeding and 0.01% for fairs. The low density and centrality of the network resulted in high market concentration. Key states in the network were Jalisco for supply and the State of Mexico for demand, with high levels of market-information loss. The main animal welfare concern identified was associated with transport duration and conditions. Although the findings comply with current legislation on pig transport duration, future measures should target improvements in transport conditions, such as implementing resting periods and enhancing traceability.

## Introduction

In order to reduce costs, enhance biosecurity, and improve productivity, pigs are moved among farms during various production stages. In Mexico, authorities classify these movements into four categories: slaughter, fattening, breeding, and transport to fairs and exhibitions. Understanding swine movement is crucial for identifying pathogen risks and commercial opportunities. Additionally, tracking swine movements enhances transparency in the food chain and supports animal welfare, addressing consumer concerns about rearing conditions [1]. Like the US, Mexico lacks all-inclusive livestock tracking systems, unlike Europe [2]. Swine movement data in Mexico is collected by SENASICA when pigs cross state borders or move within municipalities.

Social Network Analysis (SNA) has been used to study different topics related to the movement of livestock products and by-products, with a particular emphasis on animal health. Additionally, SNA studies can include both the attributes of individuals and the social relationships between them [3]. According to network theory, market participants are producers and consumers, with attributes including movement volume, frequency, intermarket distance, and the purpose of movement between source and destination.

Upon studying the pig-breeder-to-slaughterhouse network, it was found that the communities involved are distant from each other, thus preventing diseases from spreading [4]. Historically, biosecurity measures have been mainly focused on the farm level, failing to take into account the important role played by pig traders in linking farmers and consumers [5]. In the case of China, the elimination of counties with higher levels of mediation or proximity led to reductions in the size of potential epidemics [6]. Furthermore, animal welfare during transportation has become a significant public and governmental concern, leading to policies focused primarily on transport conditions and duration [7]. The Federal Veterinary Service (Servicio Nacional de Sanidad, Inocuidad y Calidad Agroalimentaria, SENASICA) regulates animal movements in Mexico. Although SENASICA can impose sanctions for noncompliance reported by third-party certification organizations [8], the supervision itself is delegated to these external entities. Current regulations limit continuous transport duration to a maximum of 20 hours, requiring at least one hour of rest after every eight hours of uninterrupted travel [9]. Training courses available for veterinarians involved in transport primarily emphasize good management practices and disease control [10], with limited attention given to animal welfare and environmental impacts.

Pork is not only part of the assortment of animal proteins consumed by Mexicans, but also an important feature of Mexican culture, in which the social relationship between pigs and human beings transcends the merely commercial, with close links being forged between pork producers on the one hand and, on the other hand, pig-breeding centers, seasons, religious festivities, and fairs [11]. Although these links give rise to mechanisms and dynamics that are hard to grasp if one is not familiar with the formal production-commercialization channels, the Mexican databases bear witness to them.

The importance of pork on the international market is determined by its financial value. In 2021, world pork trading amounted to USD 36.2 thousand million ― i.e., 28.4% more than in 2011; Spain with 17.7% of total world trade, and the USA with 16.1%, were the main exporters; China and Japan were the main importers, with 23.7% and 11.8% of all imports, respectively. A strong increase in trade with China was also observed [12].

Pork is the second most consumed meat in the Mexican market after chicken, with large amounts of resources being destined for its production [13]. Mexico is a net pork importer, and the official data show that, in the customs-tariff categories that make up the chapter on processed and refrigerated meats alone, pork imports amounted to USD 1.85 thousand million in 2021; this trend toward dependence on imports of foreign meat has grown to the point where some authors report that over 50% of the pork currently consumed in Mexico is produced abroad [14,15].

While Mexico has significant pig stocks, these do not suffice to meet the demand of pork. In 2022, national pig stocks amounted to 19.2 million heads, while 20.8 million heads were slaughtered, with a volume of 1.7 million tons of carcass and a value of MXP 87.2 thousand million pesos. Carcass efficiency was 79.2%, live pigs were sold at a cost of MXP 33.28 per kg, and MXP 50.40 per kg of carcass [16]. Moreover, in 2021 Mexico consumed just over nine million tons of meat, 41% of which was chicken, 40% pork, and 19% beef, with a total meat consumption of 18 kg per capita [17].

Mexico has an important pig-slaughtering infrastructure, with an installed capacity of 1.7 million heads per month. While just 51.7% of this capacity is used, there is more inefficiency in both municipal and private slaughterhouses (53.5%), compared to Federal Inspection Type (Spanish acronym: TIF) establishments (37.8%) [18]. This means that Mexico has enough infrastructure to slaughter sufficient pigs to eliminate its meat deficit, which stood at 565 thousand tons in 2020 [17].

Boom periods and periods of crisis and recovery can be identified over time in the Mexican pork sector [19], where there is heavy dependence on the foreign market [14] due, on the one hand, to the disappearance of a large number of pig farms, and, on the other hand, to the consolidation and concentration of production [20]. According to the Mexican Meat Council (Consejo Mexicano de la Carne, its name in Spanish) [17], there is a growing pork consumption trend in Mexico, with an average annual growth rate of 2.45% recorded between 2015 and 2019, with increasing concern about how animals are raised, transported, and slaughtered [21].

Previous studies have analyzed various aspects of Mexico’s pork production systems, including inventory, production and consumption volumes, resource availability, and pricing. We hypothesize that Mexico’s swine movement network is centralized around a few key states, potentially influencing resource allocation, environmental safety, and animal welfare during transport. This research aimed to identify the structure and movement patterns within Mexico’s live-pig market through a network-based approach.

## Materials and methods

The research used information from SENASICA [8] about swine movement between municipalities for slaughter, fattening, breeding, and fairs from 2017 to 2021. The database used contains 1,162,851 official Mexican permits for the movement of pigs across Mexico.

This study used UCINET software (version 6.27) to analyze, visualize and calculate network metrics. The data were set out in an adjacency matrix (A_ij_) whose rows pertained to the source municipalities (a_s_) and columns to destination municipalities (a_d_). The A_ij_ elements were then converted into a dichotomous adjacency matrix, with a score of 1 being assigned if adjacency existed (a_ij_ = 1) and a score of 0 being assigned if it did not exist (a_ij_ = 0). Adjacency meant that a municipality sent pigs to another one that received them. The state-level adjacency matrix (E_ij_) and the state-level dichotomous adjacency matrix were created in the same way.

The structure and relationships within the pig movement network were analyzed using the SNA method [22], with both structure and composition variables being utilized in the study. The structural variables pertained to the category of swine movement (slaughter, fattening, breeding, and fairs) between source and destination municipalities, while the composition variables pertained to the number of pigs transported per different category. The respective nodes were the pig-source and-pig destination municipalities, with the inter-municipal flows of pigs forming the links and the intra-municipal flows of pigs forming the loops.

The SNA consisted in gathering, systematizing, counting, and analyzing the number of pigs moved per source municipality and state, and per destination municipality and state, in the Mexican market. The national swine movement network or market consists of 2,446 municipalities and 32 possible state markets, so that the SNA revealed 5,982,916 possible municipal adjacencies and 1,024 possible state adjacencies. The municipalities and states are the main players in the network, and the links between them are pig shipments. Given the high market concentration, the market analysis was mainly carried out at the state level.

The elements of A_ij_ were the sources (A_i_) and destinations (A_j_) of the transported pigs, while the relationship or link was the number of pigs moved from a source to a destination (A_ij_ > 0). The importance of the network’s elements was calculated using C_i_ centrality measurements for sources and C_j_ centrality measurements for destinations, and (D_ij_) measurements for network density. The movement of pigs between municipalities (A_ii_) made it possible to use the elements of the main diagonal in the analyses.

Network density measures the proportion of actual connections relative to all possible connections within the network, taking into account both source and destination municipalities and states. It reflects the degree of interconnectedness and its impact on network cohesion and the efficiency of information transmission [23].

Network density (D_ij_) is the quotient of the number of links or pig shipments (L_ij_) divided by the number of possible links (Ai * Aj). Swine movement between municipalities and states, used in the calculations, signifies trade between municipalities and states. Density takes on 0 ≤ D_ij_ ≤ 100 values, where zero means no trade and 100 means that all the markets in the network engage in trade.


$$D_{ij}=\frac{L_{ij}}{A_{i}*A_{j}}*100$$

Average network centrality (C) was obtained by adding up the number of links for each network element (c_i_ = supply, c_j_ = demand). Market centrality is a measure of the importance of an entry market (i) or exit market (j) in the national swine movement network.


$$CR=\sum_{i=1}^{n}a_{ij}(p_{i},\;p_{j})$$

A fully connected network means CR = N = 100%, where all the elements of the network have interchange or adjacency relationships with each other. The main diagonal of the matrix was used in this study because pigs were moved within each state, while the centrality of each element of the network (CS_i_ = centrality of the state’s supply i and CD_j_ = centrality of the state’s j) was determined in the same way:


$${CS}_{i}=\sum_{i=1}^{m}a_{ij}(p_{i},p_{j})\;;\;{CS}_{j}=\sum_{i=1}^{m}a_{ji}(p_{i},p_{j})$$

The maximum centrality for each element of the supply and demand was 31, given that there are 32 supply states and 32 demand states. Such centrality means that a supply state had interchanges of pigs, or pig-related business relationships, during the period studied.

The eigenvalue proposed by [24] was used to determine the prestige of the network’s elements, based on the assumption that a network element can have the greatest centrality and density, but not the greatest prestige.

The financial structure of the swine movement network in Mexico was analyzed following the theory of economic location. The coefficients of location (Q_s_) and of specialization (Q_r_) are two relative measurements used to study the financial behavior of productive activities. Q_s_ indicates the part played, both in the region (year) and in the national market, by a given sector (slaughter, fattening, breeding and fairs); Q_si_ > 1 indicates relative specialization, Q_r_ indicates the degree of similarity of the region’s economic structure to that of the country; Q_r_ = 1 indicates greater specialization, and Q_r_ = 0 indicates intraregional diversification.

### Ethics statement

The Ethics Committee of the Universidad Autónoma del Estado de México (Mexico) approved the research protocol. Due to the nature of the study, no animals were used to obtain data.

## Results and discussion

In 2017–2021, an average of 33.3 ± 13.1 million pigs were transported annually in the national market: 87.29% for slaughter, 12.20% for fattening, 0.50% for breeding, and 0.01% for fairs and exhibitions.

Excluding fairs and exhibitions, the other swine movement categories had positive annual growth rates. Average annual inter-state pig movements formed 62.3 ± 6.6% of all mobilizations and intrastate movements formed 38.4% of them, the former dropped to an average yearly rate of 4.2% and the latter increased by 10.8%. The linear correlation (p < 0.05) was significant for slaughter, fattening and breeding, but not for fairs and exhibitions (Table 1).

**Table 1:** Classification system for swine movement in Mexico.

Year	Slaughter (%)	Fattening (%)	Breeding (%)	Fairs and exhibitions (%)
2017	17.2	10.9	11.9	28.8
2018	18.5	18.3	17.6	27.1
2019	20.7	27.8	22.9	15.1
2020	22.3	23.4	22.2	10.1
2021	21.3	19.6	25.5	18.9

Source: Author-produced using data from SENASICA.

To our knowledge, this research is the first to analyze the structure of inter-state and inter-municipal swine movement in Mexico for slaughter, fattening, breeding, and fairs. A similar study was carried out in Mexico, focused on livestock movement and effective epidemiological surveillance and disease control [8]. However, the study lacked an analysis of the structure of the swine movement network.

The high level of these movements is driven by the need for slaughterhouses with sufficient installed capacity. Official data shows that 36.9% of municipalities have a municipal slaughterhouse, 6.2% have a private slaughterhouse, and 4.9% have a TIF slaughterhouse; with average capacity utilization at 53.2% in pork-specialized slaughterhouses and 42.1% in non-specialized ones.

**Fig 1:**
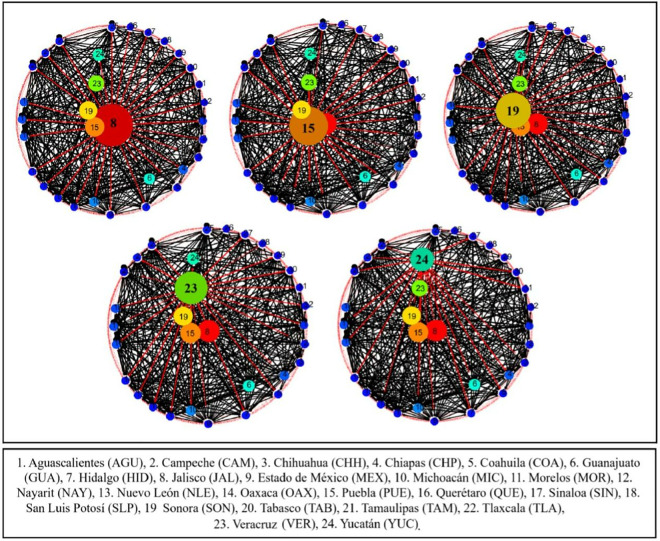
Source inter-state swine movement network degree centrality in Mexico, 2017-2021. Circle size indicates the degree of centrality of each state in the network.

**Fig 2:**
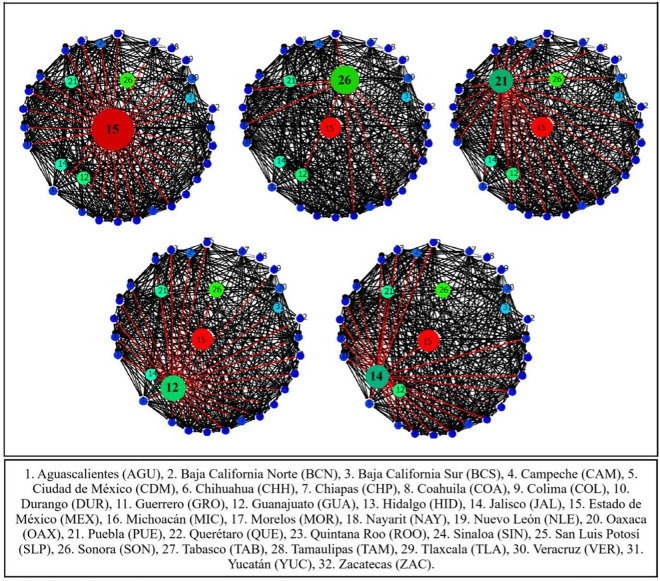
Demand inter-state swine movement network degree centrality in Mexico, 2017-2021. Circle size indicates the degree of centrality of each state in the network.

Figs 1 and 2 illustrate the end-to-end transportation network for pigs within Mexico. Locations, travel distances, and estimated speeds were calculated based on available data and communications with logistics coordinators (personal communication, FEMC). The resulting estimated transport durations remained below the maximum of 20 hours established by current legislation. Shorter pig movements between municipalities also remain within mandatory transport duration limits. Such movements typically involve smaller trucks and fewer animals, thus reducing overall transport and processing times, especially when lairage conditions and schedules are adequately coordinated with slaughterhouses. However, official public information regarding pig movement conditions in Mexico is limited. Although transport durations may be under the 20-hour maximum, legislation requires a mandatory one-hour rest period under shade after eight continuous hours. How this requirement is implemented and verified remains unclear. Third-party certifications often focus on specific sites (e.g., farms, processing plants) and may not evaluate fully integrated systems or complete end-to-end pig production flows. This limitation could hinder comprehensive animal welfare assessments and enforcement of legislation.

### Supply

While a high level of pork-production concentration was found at the state level in Mexico, 78.1% of the country’s states supplied pigs in the period studied, with 73.4% of the animals being concentrated in four states: Jalisco 22.5%, Puebla 19.4%, Sonora 17.6%, and Veracruz 13.9%. With regard to the different swine movement categories, 24.7% of the pigs transported for slaughter were concentrated in Jalisco, 21.2% in Puebla, 15.2% in Veracruz, and 13.5% in Sonora; 47.1% of the pig movements for fattening were concentrated in Sonora; 30.9% of the moved pigs for breeding were concentrated in Zacatecas, 28.2% in Sonora, 12.2% in Guanajuato and 14.9% in Nayarit; 42.2% of the pigs transported for fairs were concentrated in Guanajuato, 27.2% in Michoacán, and 19.3% in Zacatecas.

Although pigs are bred throughout Mexico, their supply was concentrated in the center of the country and 21.9% of the state markets did not supply pigs for any of the four swine movement categories analyzed. Seven regions were formed based on the annual average of pigs transported, but none had contiguous states. The three most important states (Sonora, Jalisco, and Puebla) are strategic: Sonora borders with the USA, Jalisco is strategic due to its highly developed infrastructure, and Puebla borders with the country’s biggest markets: Mexico City and the State of Mexico.

### Demand

Pig demand involved an average of 167 of Mexico’s municipalities and all states, with its distribution being even more heterogeneous than that of supply. The State of Mexico accounted for 29.5% of all pig production, followed by Sonora at 15.5%, Guanajuato at 11%, and Puebla at 9.9%. Puebla and Sonora markets grew by 188.2% and 10.3%, respectively, while market growth in the State of Mexico and Guanajuato was negative with −5.3% and −1.5% respectively. Among the remaining markets are Tamaulipas and Chihuahua, with respective growth rates of 155.3% and 81.0% but low levels of market share, while Sinaloa, with a low market share, grew by 78.9%.

Market share per type of market was uneven, with the biggest share being concentrated in the country’s center. Four states concentrated 69.0% of the pigs transported for slaughter: three located in central Mexico (Guanajuato, Puebla, and the State of Mexico) and one in northwestern Mexico (Sonora). Similarly, 74% of the pigs transported for fattening were concentrated in four states: two northwestern states (Sinaloa and Sonora), and two central states (Jalisco and Guanajuato). 72.3% of the animals destined for breeding were also concentrated in four main markets, with the two main locations being the central states of Jalisco and Guanajuato. While 70.4% of pigs destined for fairs and exhibitions were concentrated in four markets: three central states (Michoacán, Jalisco, and Guanajuato) and one southwestern state (Veracruz).

### Networks

Fig 1 shows the structure of the national swine movement network, which, on average, consisted of 25 source states and 32 destination states (Fig 2). No supply of pigs was found in just over one-fifth (21.9%) of the source states. However, demand for them did exist in such states.

The network’s average annual density for all four categories of pig movement was 25.6 ± 7.2%, with a 2.2% average annual growth. The highest density (30.1 ± 2.0%) was for slaughter, and the lowest (15.9 ± 2.1%) was for fairs. The average annual centrality for all the movement categories was 40.6 ± 2.1%, with transport for slaughter having the highest density (29.6 ± 2.0%) and transport for fairs the lowest (5.2 ± 0.8%).

Regarding supply at the state level, Jalisco had the highest degree of supply centrality, having links with 100% of the states, followed by Sonora with 96.9%, and the State of Mexico with 90.6%. However, the most influential element of the network was that about to the most valuable markets for pigs sent, pigs received and links. Such valuable markets were identified with the eigenvalue, with an average eigenvalue of 0.19 for all the source states. Jalisco was the most influential state with 0.29, followed by Sonora and the State of Mexico with 0.28 and 0.27 respectively.

In addition to demand at the state level, the State of Mexico had the highest degree of demand centrality, having links with 96.0% of the source states and receiving pigs from 24 of the 25 source states. Other important destinations were Jalisco, Puebla, Veracruz and Guanajuato, which received pig shipments from 22 sources. The State of Mexico was the most influential destination with an eigenvalue of 0.25, while the other four most important markets had an eigenvalue of 0.23.

### Municipal

**Fig 3:**
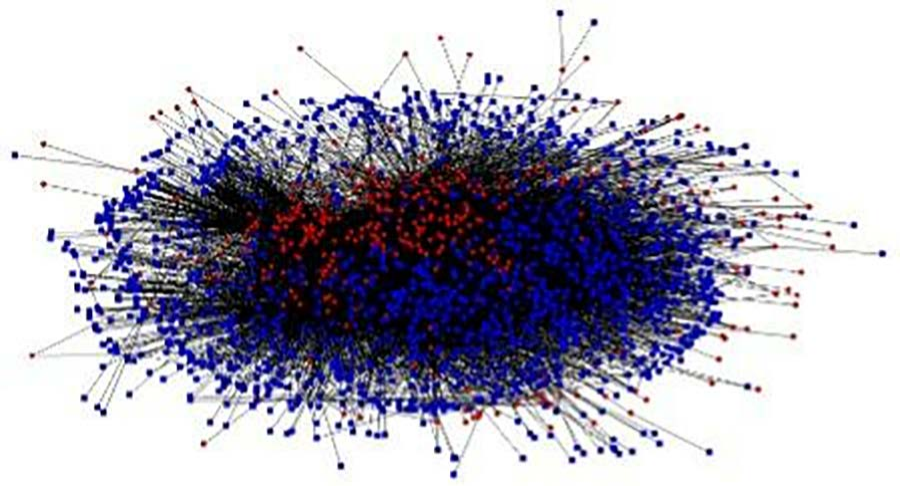
Inter-municipal swine movement network degree centrality in Mexico, 2017-2021. Red = supply. Blue = demand.

The structure of the national network for all categories of swine movement was complete. Of the country’s 2,446 municipalities, only 6.8% participated in pig supply and only 15.3% in pig demand; 16.0% of the municipalities participated in slaughter supply, 6.9% in fattening supply, 3.7% in breeding supply, and 0.8% in fair supply, while 26.9% participated in slaughter demand, 20.9% in breeding demand, 12.4% in fattening demand, and 1.0% in fair demand. Network density was very low, with only 12,355 of the 582,426 potential municipal links materializing, representing a density of 2.1% (Fig 3).

The average exit density for all the categories and years was 31.4 of 1,482 possible links; average entry centrality was a quarter of exit centrality, with only 8.3% of 393 possible links materializing.

Municipal-supply concentration was high, with 41.3% of all transported pigs being concentrated in 10 municipalities: 13.9% in municipalities in Jalisco, 11.5% in municipalities in Sonora, 9.9% in municipalities in Veracruz, and 5.9% in municipalities in Puebla. Although the municipality of Jalacingo in Veracruz represented 7.0% of all the pigs for the four categories of swine movement, it had links with only 3.5% of the destinations. Lagos de Moreno in Jalisco and Cajeme in Sonora, both with 5.6% of all swine movements, being linked with 13.8% and 12.0% of the destinations respectively. The most important municipalities in terms of centrality were Tepatitlán de Morelos in Jalisco, Nopaltepec in the State of Mexico, and Degollado in Jalisco (24.0%, 21.8% and 19.8% of all the transported pigs). However, their combined market share was only 2.6%.

The concentration in municipal demand was even higher than in municipal supply, with 54.5% of all the pigs being concentrated in 10 municipalities: 19.2% in municipalities in the State of Mexico, 14.2% in municipalities in Sonora, 8.3% in municipalities in Guanajuato, 7.7% in municipalities in Puebla, and 5.0% in municipalities in Yucatán. The municipality with the most centrality was Cuautitlan in the State of Mexico, which was linked to 38.2% of the source municipalities, followed by Pénjamo in Guanajuato with 34.4%, and La Paz in the State of Mexico with 32.1%. One fifth of all the pigs in demand for all four categories of swine movement, was concentrated in these three municipalities.

### National swine movement: implications for animal welfare and environmental impact

The centrality and density measures show a concentrated national network where information does not flow to all elements of the network. The loss of information in national supply and demand for all categories of swine movement and for the analyzed period was 40.3% and 53.3% of the markets, respectively. The states of Jalisco and the State of Mexico lead the country’s supply and demand for pigs, respectively. The former is due to its relationship with all markets, and the latter is due to the volume received. While land transportation is a critical tool for the pig supply chain, it also represents a risk factor for the introduction and spread of infectious diseases in farms [25]; in the case of Mexico, a concentrated production favors disease control through increased mechanization and rapid diagnostics of frequently occurring diseases [26], particularly in geographically isolated areas like the Yucatan Peninsula with limited road access, and Sonora with an arid climate which help limit pathogen spread. However, special attention must be given to animal welfare during pig movement, as factors like journey duration, extreme temperatures, feed withdrawal, and lack of water can compromise both pig welfare and carcass quality [7].

An analysis of the movement of 60.65 million pigs in Brazil, from 2015 to 2017, was conducted to target disease surveillance at the municipal level [27]. The study evaluated both static and temporal pig movement networks, concluding that static models may overestimate outbreak size, particularly for rapidly spreading diseases. As a result, they recommend temporal analysis, which more accurately reflects the chronological nature of pig movements. It is important to acknowledge that until other factors, such as the movement of feed trucks and short-distance transport of gilts or culled sows, are considered, our understanding of disease transmission pathways will remain limited [28].

Transporting pigs for fattening can lead to economic losses due to mortality, skin damage, deterioration of meat quality [29], lower meat pH, reduced water-holding capacity, and increased drip loss [30]. In a study conducted in the USA, [31] assessed the impact of transportation on pig mortality, non-ambulatory pigs, and total losses, reporting rates of 0.26%, 0.63%, and 0.88%, respectively. According to the authors, seasonal temperatures are a key factor in swine movement, with the highest mortality being observed in July (0.29%), August (0.32%), and September (0.30%), while the lowest rates occurred in February, March, and April (all 0.22%). They also found that more pigs were Dead On Arrival (DOA) during the summer compared to cooler months, with DOA rates increasing with temperatures between 70°F to 100°F [32].

Distance traveled affects the health and quality of livestock destined for slaughter and reduces the profitability of the production chain. However, large slaughterhouses determine the commercial conditions. While transporting carcasses instead of live pigs could be more profitable in Mexico, animal products and subproducts have diverse marketing channels, processes, and uses after slaughter. When correlating consumption and income with the type of pork cut and place of purchase, people prefer pre-cut, unpackaged meat and to buy it in butcher’s shops and markets because it is neither frozen nor refrigerated [33]. Additionally, increased transportation of live pigs from farm to slaughterhouse increases environmental burdens [34]. Production centers are supported by specific physical, biological, infrastructural, and strategic economic factors. The northeast and southeast regions of Mexico, for example, benefit from access to lower-cost feed inputs from the USA and the ease of exporting products. These regions have specialized in pork production since 1994 [35]. Moreover, the implications are not only economic but also have significant environmental effects. According to [36], the origin of feed inputs is a major contributor to increased environmental impacts, particularly in the Fossil Depletion (FD) category. Acquiring inputs from a distance of 900 km resulted in a fivefold increase in the FD category, while a ninefold increase was observed from a distance of 1800 km, compared to a base scenario of 400 km.

Swine movement requires detailed monitoring of environmental conditions, especially in states with extreme weather conditions, such as Sonora, Yucatan, and Veracruz. In addition, road condition surveillance is necessary to prevent and control long transport duration as much as possible from supplier states. Similarly, transport and lairage conditions should be overseen in states receiving significant volumes of animals.

Average daily swine movement frequency is low in Mexico (24.4 ± 39.4), but high in Jalisco (141.9 ± 8.7) and in Puebla (117.7 ± 14.3). The daily frequency of pig movements in the USA is 100 [37], being higher than the average in Mexico, but lower than in Jalisco and Puebla. With regard to information, Jalisco and Puebla have greater average data-management capacity than the USA. In the case of Mexico, pigs for slaughter have the highest movement frequency, while pigs for fattening have the highest movement frequency in the USA [38]; this means that pig farms in the USA have a slaughterhouse linked to them, while those in Mexico do not. This raises animal welfare and economic concerns due to the stress of transport and its impact on meat quality. Contrary to what might be expected, Puebla and Jalisco do not experience bottlenecks due to low installed capacity in their slaughterhouses [18,39]. Mexico has 1,175 animal slaughtering facilities, 70% of which specialize in pig processing. These have an installed monthly capacity of 1.7 million heads, currently operating at 42.1%. The top five pig-producing states account for 34.2% of the total capacity, with the primary destination state (State of México) comprising 24.7%. Additionally, federally inspected slaughterhouses (Tipo Inspección Federal, TIF), although representing only 10.2% of total facilities, account for 81.3% of the installed capacity and operate at 46.8% utilization.

The present study identifies Cuautitlan and the State of Mexico as the regions with the highest demand for all swine movement categories, despite representing 0.44% and 13.5% respectively, of Mexico’s 2020 population which stood at 126 million [40]. However, their geopolitical and socioeconomic significance as part of the Valley of Mexico, with a population of 21.8 million is notable, linking Mexico City and 60 neighboring municipalities [41]. Animal welfare is particularly concerning in this region, given the large numbers of pigs transported daily from states with large swine populations, such as Jalisco, Puebla, and Veracruz. Mexican law mandates lairage at slaughter plants and a 20-hour transport limit [42], which should be feasible based on farm-to-slaughter distances analyzed. However, this study identified swine transport exceeding 8 hours, particularly from northern states to the country’s center. Mexico lacks government-approved animal welfare certifications to ensure proper handling and transport. Additionally, there is no public data on economic losses from poor meat quality due to handling and transport conditions. Nonetheless, this study identifies opportunities for producers and local authorities to improve transport quality and animal welfare. Practical interventions could include traceability programs supported by governments (e.g., Pig Tracker in Europe [43]) or industry-led initiatives (e.g., PigTRACE in Canada [44]) to ensure compliance with national and international legislation, such as the standards outlined in the Terrestrial Animal Health Code of the World Organisation for Animal Health. Another area of opportunity involves developing and implementing guidelines, training programs, and certification schemes focused on animal welfare and production quality across various stages of pig production. Examples include industry-led initiatives such as Pork Quality Assurance and Transport Quality Assurance by the U.S. National Pork Board, and Animal Handling Guidelines by the North American Meat Institute (NAMI), as well as programs led by government agencies or non-governmental organizations.

Overall, the movement of pigs to slaughter, fattening and breeding markets has increased. However, the fattening market has shrunk by 9.2% in the last 2 years, possibly due to the Covid-19 pandemic, which most prejudiced the fairs-and-exhibitions market, causing a reduction of 34.4%. The growth of the breeding market should be stressed, since it might imply an increase in national productive and multiplier capacity, which could be a strategy adopted by Mexico aimed at reducing the deficit in pork [14] and satisfying the 13.4% increase in per-capita demand that has occurred over the last 5 years; likewise, it might be a strategy for reducing the instability in the market stemming from pandemics (COVID-19), animal health issues, rising input costs, or a response of pork producers to market signals.

The asymmetry among the main source municipalities, which accounted for 34.3% of all pig movements in Mexico, is largely due to various factors, with transportation infrastructure for moving inputs and products being the most important. Jalacingo, the main source municipality, is located 176 km and 2.5 hours from Mexico’s main port in Veracruz. Lagos de Moreno and San Juan de los Lagos, which are the second and fourth, respectively, most important source municipalities, and are located 498 km from the ports of Manzanillo, Puerto Vallarta, and Lázaro Cárdenas. Cajeme and Guaymas (the third and fifth most important municipalities) are situated at the port of Guaymas. In addition, all above mentioned municipalities have direct access to Mexico’s railway and highway network. For example, Jalacingo in Veracruz is located 85 km or 1.15 hours from its main destination in east Puebla. Lagos de Moreno in the state of Jalisco is 165 km or 3.1 hours from Pénjamo in Guanajuato. The municipality of Cajeme in Sonora is 226.6 km or 3.5 hours from the municipality of Ahome in Sinaloa. San Juan de los Lagos, which is Jalisco’s main destination market, and Guaymas in Sonora located 129.1 km or 1.43 hours from Cajeme, Sonora.

Based on the economic location theory, it was possible to elucidate the financial structure of the swine movement network in Mexico. All the variables that determine the location and destination of pigs focus on two measures: the coefficient of localization (CL) and the coefficient of specialization (CS).

Although the average CL of 1.01 showed that all the types of swine movements were self-sufficient, this was not the case for fattening and breeding. The average CS for all the types of swine movement was low (8.8%), while the lowest specialization coefficient pertained to slaughter and the highest one to pigs for fairs, which are diversified production systems. The results point to a diversified pork production that leads to a low self-sufficiency level (Table 2).

**Table 2:** Spatial concentration per different categories of swine movement between 2017 and 2021.

Region/ year	2017	2018	2019	2020	2021	CE
Slaughter	1.05	1.00	0.96	0.99	1.01	0.010
Fattening	0.66	1.00	1.30	1.04	0.92	0.072
Breeding	0.72	0.96	1.07	0.99	1.19	0.055
Fairs	1.77	1.50	0.70	0.45	0.86	0.215

Source: Author-produced using data from SENASICA.

### Exportation trade

In addition to satisfying the national pork demand, Mexico has an active role in the global pork trade that involves swine movements to exportation regions. Nowadays, the USDA has authorized 124 Mexican centers for processing and exporting pork products to the USA [45]. The value of Mexican pork exports has grown from zero in 1991 to USD $772.8 million in 2023. According to [46], shipments from Sonora (northwest Mexico) can reach Asian ports via Long Beach, CA, on the U.S. West Coast, in approximately 18–20 days. It takes about three weeks from processing plants to custom clearance, ensuring that the freshness of Mexican pork is maintained as well as or even better than other North American pork products. For frozen meat, most shipments are made from Mexican ports, primarily Ensenada or Manzanillo, taking 20–23 days due to their lower costs.

In the states of Yucatan (southeast) and Sinaloa (northwest), shipments primarily consist of frozen products. Land transport is used to reach the ports of Manzanillo, Lázaro Cárdenas, and Mazatlán, where the products are then shipped. On average, shipping takes 20–23 days. From Jalisco, land transport is used to reach the ports of Manzanillo and Colima, with the journey also taking between 20 and 23 days to reach the destination. Some producers with exportation trades have implemented animal welfare certifications by non-governmental agencies (third party) that include transport criteria [47].

### Study limitations and recommendations

Regarding limitations of the study, the analysis lacked detail about farm-level data, seasonal impact, and evaluation of animal characteristics, such as breed, weight, age, and total mortality all of which could confer technical and competitive advantages among farms. Additionally, factors such as the effects of the Covid-19 pandemic on the supply chain were not fully explored, along with other variables that are critical for gaining a comprehensive understanding of Mexico’s pork production systems.

The research aimed to collect official data from municipalities about four categories of swine movement over a five-year period, revealing widespread pig production nationwide. However, specific production areas were not identified; instead, they were inferred based on the volume of swine movement. The data capture pig movements without distinguishing the scale of origin farms (large, medium, or small-scale). However, the daily resolution of the records could allow for analysis of seasonal trends and production patterns throughout the year.

The findings of our study could aid national and global markets make better business choices, encourage new research, and shape public policies. Currently, livestock production systems worldwide are under significant pressure, especially regarding environmental impact, health management, and animal welfare. Future studies must focus on the importance of the Mexican pork industry in ensuring food security.

In Austria, a general reduction in the number of pig batch movements and pig farms was observed, despite an increase in the total number of pigs transported [48]. The authors link this trend to system intensification and the disappearance of small-scale farms, a pattern also seen in Mexican pig farming. The analyzed swine movement networks by [48], also showed fewer movements but with larger pig batches. Furthermore, the fragmentation of pig farming systems in countries like Switzerland has been reported to reduce the likelihood of infectious disease spread [49].

The importance of using SENASICA’s swine movement records goes beyond being official information; it also provides vital Mexican data for monitoring and, in the event of an outbreak or epidemiological crisis, for implementing necessary containment protocols. However, local swine movements in Mexico, primarily from short production chains involving small-scale pig farms are known to occur without registration, but their extent remains unquantified.. These production units accounted for up to 30% of pork and 40% of the national pig inventory, involving approximately 20,000 producers, primarily located in rural municipalities engaged in small-scale production [50]. These types of pig farms have access to four market outlets; the first market is direct sales to consumers, the second is direct sales to retailers, the third market is a direct sale to commission agents, and the fourth market outlet is the sale through farmer associations [51]. According to official data, these types of farms account for just under 30% of the total pig inventory, operating within short marketing circuits, but the process is not entirely equitable since the butcher or processing plant buys live pigs directly from producers, ultimately dictating the purchase price.

## Conclusions

Via social-network analysis, it was possible to determine the movement and structure of the live-pig market in Mexico. The structure of the national swine movement network has remained unchanged, with 25 source states, 32 destination states, and 4 categories for swine movement. It is the structure of the pigs-for-slaughter category that has underpinned the national structure, while the numbers of both source states and destination states have been the same, thus allowing a high concentration of production and consumption. The centrality values of the leading states and municipalities attest to an important capital asset where there is not only a flow of business information but also a concentration of power and dissemination. The inferred structures imply the concentration of resources and infrastructure required for pig production and distribution, highlighting the need to leverage market-specific structures to optimize the flow of livestock, information, and pig supply. Now that the said structures have been identified, it is possible to develop proposals for animal welfare, health, and production improvement and specialization where feasible, and also to take advantage of Mexico’s most important state market, Jalisco. Public policy should focus on optimizing available resources, particularly slaughterhouse infrastructure, to enhance the competitiveness of states with the highest concentration of pig supply and demand.

## Supporting information

S1 Data Dataset (XLSX)
